# Opportunities to Increase Influenza Vaccine Uptake Among Pregnant Women: Insights from Surveys in 2013 and 2023

**DOI:** 10.3390/vaccines13060589

**Published:** 2025-05-30

**Authors:** Yuanyuan Zhang, Wanting Hong, Rui Wang, Lin Bao, Cheng Liu, Pengwei Cui, Yayun Tan, Hui Hang, Yuanyuan Pang, Qian Xu, Ge Tian, Jiarun Jiang, Suping Zhang, Liling Chen

**Affiliations:** 1 School of Public Health, Nanjing Medical University, Nanjing 211166, China; yuanyuan_zhang2025@163.com (Y.Z.); wanting_hong@stu.njmu.edu.cn (W.H.); 2 Suzhou Center for Disease Control and Prevention, Suzhou 215123, China; 302110120952@stu.xzhmu.edu.cn (R.W.); baolin19870304@163.com (L.B.); szcdclc@163.com (C.L.); xiaoxiaowei90@126.com (P.C.); helloyayun@163.com (Y.T.); szcdchanghui@126.com (H.H.); pyyyubingxi@sina.com (Y.P.); qianxu_0526@163.com (Q.X.); getian_8725@163.com (G.T.); spzhang012@163.com (S.Z.); 3 School of Public Health, Xuzhou Medical University, Xuzhou 221004, China; 303110120949@stu.xzhmu.edu.cn

**Keywords:** influenza, influenza vaccine, pregnant women, knowledge, attitude and practices (KAP), vaccination coverage

## Abstract

Background: Health departments disseminate health education related to influenza to the public through various media in China. We examined knowledge, attitudes, and practices regarding influenza and the influenza vaccine (KAP-flu) over time among pregnant women (PW) compared to non-PW. Methods: A cross-sectional survey was conducted in Suzhou, China in 2013 and 2023. We included and interviewed PW seeking prenatal care and excluded PW there for non-routine visits. The comparison group was drawn from non-PW seeking physical examinations at the same facilities. Stratified cluster sampling was used to enroll participants from the various levels of prenatal-care facilities. Results: In 2013, we surveyed 1673 PW and 401 non-PW, and in 2023, we surveyed 2195 PW and 1171 non-PW. The proportion of PW who had ever heard of the influenza vaccine showed no significant change, at 56% in 2013 and 57% in 2023; by contrast, there was a significant increase among non-PW (55% to 78%). The proportion of pregnant participants who knew when to get vaccinated dropped from 14% to 12%, in contrast to the increase among non-PW (6% to 20%). The proportion of PW who believed that the influenza vaccine is effective dropped from 91% in 2013 to 76% in 2023, in contrast to the stable value among non-PW (84% to 82%). In 2023, pregnant participants exhibited lower levels of knowledge about both influenza disease and the influenza vaccine, along with less positive attitudes toward the effectiveness and safety of the vaccine. They also showed lower willingness to vaccinate and lower vaccination rates compared to non-pregnant participants. Concerning KAP-flu among PW, less than half recognized that influenza is different from a common cold; fewer than one in five understood the timing and frequency of vaccination or the policy prioritizing PW for influenza vaccination; vaccination coverage remained below 2% over time. Conclusions: PW had concerning gaps in knowledge and attitudes regarding influenza and the influenza vaccine compared to non-PW in Suzhou, China. Specific actions targeting PW, such as initiatives leveraging the maternal and child healthcare system, are warranted to reduce the gaps.

## 1. Introduction

Each year, approximately 7% of pregnant women develop influenza illness and 0.2% require hospitalization, according to estimates from China from 2018–2023 [1]. Influenza can lead to severe complications in pregnant women, including increased risks of hospitalization, pneumonia, and exacerbation of underlying health conditions, while also posing significant risks to the fetus, such as preterm birth, low birth weight, and potential developmental issues [2,3]. The influenza vaccine is effective in mitigating these risks and protecting both maternal and fetal health [4]. Uptake of the seasonal influenza vaccine remains low in pregnant women despite recommendations from public health agencies to administer influenza vaccination to prevent severe disease. In high-income countries, vaccine uptake in this population varies from 5% to 58% [5], while consistently lower coverages are observed in resource-limited countries [6]. Previous studies have documented factors influencing vaccination behavior in this population, such as knowledge gaps, misconceptions about vaccine safety and effectiveness, and limited access to vaccination services [7]. However, few studies have specifically examined how pregnant women’s knowledge, attitude, and practices regarding influenza and its vaccine (KAP-flu) compare to those of non-pregnant women, particularly over time.

In China, the average influenza vaccination coverage across the entire population remains below 3% and is even lower among pregnant women [8]. Since 2014, the Chinese Center for Disease Control and Prevention has issued an annual technical guideline recommending the inactivated seasonal influenza vaccine for high-risk groups, which include pregnant women, the elderly, children under five years old, and healthcare workers [9]. National and local health departments disseminate health education to the public through various media channels such as television (TV) programs, WeChat (the most popular social communication tool in China), and websites. The educational content typically addresses topics such as like what influenza is, its potential harms, and general prevention methods including personal hygiene, ventilation, and influenza vaccination. However, it remains unclear to what extent the recommendations of public health agencies and general health-education efforts impact pregnant women specifically. Additionally, it is uncertain whether pregnant women perceive the general health-education efforts differently than non-pregnant individuals and if and how they exhibit distinct patterns of change in their knowledge, attitudes, and practices regarding influenza vaccination. To improve the efficacy of health-education efforts for influenza prevention among pregnant women, it is crucial to identify the significant barriers they encounter, as well as this population’s unique needs in terms of information or support.

This study investigated this question situation and whether there were any differences in the changes in KAP-flu uptake between pregnant and non-pregnant women based on a survey administered in 2013 and a survey administered in 2023. The aim was to identify specific gaps and opportunities to increase uptake of the influenza vaccine during pregnancy to protect against severe outcomes.

## 2. Methods

### 2.1. Study Design and Setting

**Figure 1:**
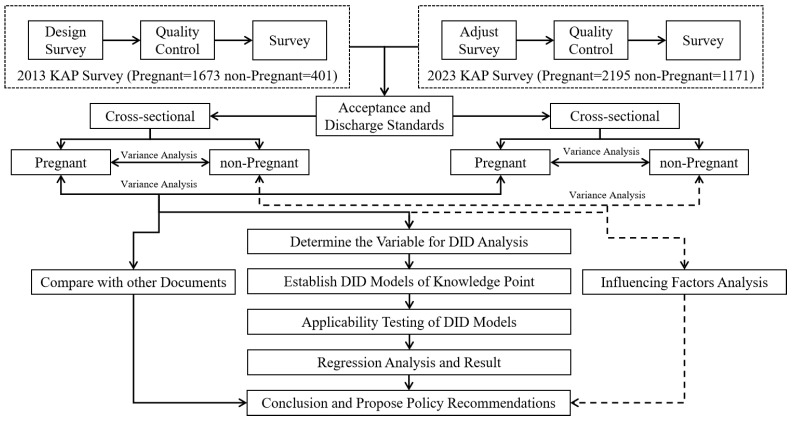
Study flow chart including the survey administered to pregnant and non-pregnant women in Suzhou, China in 2013 and 2023. KAP: knowledge, attitudes, and practice; DID: difference-in-differences.

This study analyzed the quantitative data from two separate cross-sectional surveys that utilized the same design, procedures, and data-collection tools to assess KAP-flu uptake during the influenza epidemics in 2013 (January) and 2023 (April, as influenza activity started later than in previous years) in Suzhou (Figure 1). Suzhou, located in eastern China, is one of the country’s most developed cities, with a population of approximately 16 million. Vaccine-coverage data from 2013 have been previously shared in other reports [10].

### 2.2. Participants

Pregnant women in any trimester seeking prenatal care at a designated facility were eligible for inclusion in this study. We excluded those who declined participation and those requiring non-routine prenatal care (e.g., visits due to pregnancy complications such as threatened miscarriage). Non-pregnant women aged 20–49 years seeking physical examination at the same facilities were included in the comparison group.

### 2.3. Data Collection

Trained investigators conducted face-to-face interviews with both pregnant and non-pregnant participants using the same structured questionnaire, which was designed based on the KAP theoretical model in 2013 and in 2023 [11]. The questionnaire consisted of basic information; knowledge, attitudes (feelings/thoughts/beliefs), and practices regarding influenza and its vaccine; perceptions influencing vaccination practices; ways of obtaining health information; and acceptability of potential interventions to increase vaccination. The KAP-flu items were developed based on the validated tools used in previous studies [12]. The surveys included a combination of quantitative questions, including multiple-choice questions and Likert scales (Supplement S1). 

### 2.4. Study Size

We used a stratified cluster-sampling approach to enroll participants. First, prenatal-care facilities were stratified into municipal-level, county-level and township-level facilities. Within each level, we selected prenatal-care facilities based on their willingness to participate. In each selected prenatal-care facility, we approached all visiting women at the triage points to screen them for eligibility, determine their interest in participation, and obtain their consent during regular business hours beginning on the first day. Enrollment and data collection stopped on the day when the target sample size for each level was reached. The sample size was preliminarily calculated as $n=\frac{N}{1+N{\left(e\right)}^{2}}$, where *N* = the total pregnant population seen at each level of prenatal-care facility *e*, the desired precision around outcome measures, was assumed to be equal to 0.05. We then assumed the cluster-sampling effect to be equal to 1.5 and the proportion of valid questionnaires to be 95% to estimate the final target number of pregnant participants to include in the sample. The target sample size was set at 400 for non-pregnant participants, matching the number set for pregnant participants at each level.

### 2.5. Data Analysis

We described and compared participant characteristics and KAP-flu items between 2013 and 2023 for pregnant and non-pregnant participants to contextualize the changes in beliefs and practices related to KAP-flu among pregnant participants over time. For the 2023 data, we compared the KAP-flu items and the ways of obtaining information between pregnant and non-pregnant participants to identify the most recent disparities between the two groups. In 2023, we also described perceptions related to influenza vaccination and the acceptability of various interventions aimed at increasing vaccination rates among pregnant participants. For responses collected on a Likert scale, we categorized and analyzed them as dichotomous variables. Given the low vaccination-coverage rate, we described the perceived relationship between KAP-flu factors and vaccination practices instead of modeling a relationship. Pearson’s Chi-square test was used to determine whether there was a statistically significant difference between two proportions. Mann–Whitney U non-parametric statistical tests were used to determine whether there was a significant difference between the distribution of two groups in terms of characteristics such as family size. Difference-in-differences logistic regression was used to verify the change in the outcome of interest (e.g., knowledge) over time between pregnant and non-pregnant participants, adjusting for confounding factors including age, education level, household income, family size, and underlying condition. Statistical significance was defined by a two tailed *p*-value of less than 0.05. We used R software (4.4.0 version) for all data analyses.

## 3. Results

### 3.1. Participant Characteristics

We interviewed 1673 pregnant participants and 401 non-pregnant participants in 2013 and 2195 pregnant participants and 1171 non-pregnant participants in 2023 (Table 1). Between 2013 and 2023, pregnant participants showed similar patterns of change in demographic characteristics compared to non-pregnant participants. Compared to 2013, the median age of participants in 2023 was significantly older (median 26 years in 2013 vs. 30 years in 2023 for pregnant participants; 24 years in 2013 vs. 29 years in 2023 for non-pregnant participants), more likely to be college educated (44% vs. 74% for pregnant participants; 65% vs. 88% for non-pregnant participants), and more likely to have a household income ≥ CNY 250,000 (3% vs. 27% for pregnant participants; 5% vs. 19% for non-pregnant participants). Although participants in 2023 had statistically significant differences in family-size distribution (median number of other persons living in the household: three vs. two for pregnant participants; two vs. two for non-pregnant participants), underlying health conditions (2.4% vs. 2.6% for pregnant participants; 0.3% vs. 2.4% for non-pregnant participants), and BMI before pregnancy (mean: 20.9 vs. 22.4 for pregnant participants; 20.3 vs. 21.9 for non-pregnant participants) compared to 2013, the absolute differences between medians, means, or percentages were minimal.

In 2023, pregnant participants had a significantly lower education level compared to non-pregnant participants but a higher annual income (Table 1).

### 3.2. Knowledge on Influenza Disease and the Influenza Vaccine

From 2013 to 2023, negative changes in knowledge were noted among both pregnant and non-pregnant participants (Table 2). There was a statistically significant decrease in the proportion of both non-pregnant and pregnant participants who recognized the harms of influenza to mothers. Specifically, the percentage of pregnant participants who understood that influenza could cause severe disease dropped from 80% in 2013 to 62% in 2023; the percentage of those aware that it could lead to hospitalization decreased from 75% to 65%; the percentage of those recognizing the potential for death fell from 63% to 49%. Despite some increases, less than half of pregnant participants (35% in 2013 vs. 49% in 2023) correctly identified that influenza and the common cold as different diseases; less than half pregnant participants recognized health risks to the fetus, including the risk that influenza during pregnancy could result in miscarriage/stillbirth (13% in 2013 vs. 44% in 2023) or abnormal fetal development (28% in 2013 vs. 50% in 2023). In 2023, compared to non-pregnant participants, pregnant participants were significantly less likely to know the symptoms of influenza or recognize the health risks to both mothers and fetuses.

From 2013 to 2023, there was no significant increase in the proportion of pregnant participants who had ever heard of the influenza vaccine, which remained stable at 56% in 2013 and 57% in 2023 (Table 2). In contrast, non-pregnant participants experienced a significant increase, from 55% to 78%, during the same period. Among those who ever heard of the influenza vaccine, there was a statistically significant decrease in the proportion of pregnant participants who knew when to get vaccinated in Suzhou, from 14% to 12%. Conversely, there was a significant increase in the proportion of non-pregnant participants with this knowledge, rising from 6% to 20%. A decreased proportion of both pregnant and non-pregnant participants correctly reported that the influenza vaccine is provided annually (39% vs. 33%). Although there was slight increase from 12% in 2013 to 20% in 2023, less than one in five participants responded correctly that pregnant participants were one of the priority groups who should receive seasonal influenza vaccination. In 2023, compared to non-pregnant participants, pregnant participants was significantly less likely to ever have heard of the influenza vaccine and a significantly smaller proportion of them provided accurate responses regarding the vaccine on all checked items, including the time, frequency, and place of vaccination and the treatment of pregnant women as a priority vaccination group.

### 3.3. Attitude Toward the Influenza Vaccine

Among those who had ever heard of the influenza vaccine, there was a significant decline in the proportion of pregnant participants who believed that the influenza vaccine worked well or worked sometimes, dropping from 91% in 2013 to 76% in 2023. In contrast, there was no significant change among non-pregnant participants during the same period (Table 2). In 2023, a significantly lower proportion of pregnant participants compared to non-pregnant participants believed that the influenza vaccine worked well or worked sometimes (76% vs. 80%, *p* < 0.05) or believed that the influenza vaccine was safe (69% vs. 81%, *p* < 0.05).

### 3.4. Influenza Vaccination and Non-Pharmaceutical Prevention Practices

Among all pregnant participants, the proportion of those who reported receiving an influenza vaccine in the 12 months prior to the survey remained low, with a slight increase from 0.6% in 2013 to 1.9% in 2023. However, it is worth noting that all vaccinations occurred prior to conception rather than during pregnancy. In comparison, among all non-pregnant participants, the proportion of those who had been vaccinated in the past 12 months also remained low but showed a statistically significantly greater increase (from 0.0% in 2013 to 5.3% in 2023) when compared to pregnant participants (*p* < 0.05) (Table 3). The willingness of pregnant participants who had ever heard of the influenza vaccine to accept influenza vaccination during pregnancy significantly decreased, from 9% in 2013 to 4% in 2023 (*p* < 0.001). In contrast, the willingness of non-pregnant participants to accept influenza vaccination if they became pregnant significantly increased, from 3% to 9% (*p* < 0.001). Additionally, there was no statistically significant change in vaccination coverage among family members of pregnant participants over the same period, with that value being 8% in 2013 and 7% in 2023. Conversely, there was a significant increase among family members of non-pregnant participants, rising from 1% to 13%. Overall, in 2023, both uptake of the vaccine and willingness to vaccinate were significantly lower among pregnant participants compared to non-pregnant participants.

The use of non-pharmaceutical approaches to prevent influenza transmission surged in 2023 among both pregnant and non-pregnant participants (Table 3). For instance, among pregnant participants with a family member with a fever and cough, the proportion who tried to avoid close contact increased from 25% in 2013 to 87% in 2023. Similarly, those who reported increasing their hand-washing frequency rose from 10% in 2013 to 76% in 2023, and the percentage of participants wearing masks jumped from 6% in 2013 to 82% in 2023. In 2013, there was no significant difference between pregnant and non-pregnant participants regarding the adoption of non-pharmaceutical prevention approaches, including avoid contacting with sick individuals, increasing hand-washing frequency, and wearing masks.

The differences in the above changes over time between pregnant and non-pregnant women were confirmed by multivariable difference-in-differences regressions (Supplement S2).

### 3.5. Perceptions Influencing Influenza Vaccine Uptake Among Pregnant Participants

Among participants who had ever heard of the influenza vaccine, the most frequently reported “very relevant” or “extremely relevant” reason to seek influenza vaccination among pregnant participants in 2023 was concern that influenza could cause fetal harm (Table 4). In addition to worries about the harm to fetus, the next two most frequently reported relevant reasons were concerns about harm to the mother and recommendation by a doctor.

Concerns about side effects was the main barriers to receiving vaccination among pregnant participants in 2023. Specifically, 20% of pregnant participants felt a hypothesized “severe reaction following a prior vaccination” was a very relevant or extremely relevant reason for not receiving the influenza vaccine. Additionally, hypothesized history of “allergy to the vaccine” (20%) and “concerns about side effects” (15%) were also perceived as “very relevant” or “extremely relevant” reasons for not receiving the influenza vaccine.

### 3.6. Intervention Needs

In 2023, fewer pregnant participants wanted to receive information regarding vaccination timing and location (73% vs. 78%, *p* = 0.005) or about which populations are recommended as vaccination priority populations (51% vs. 59%, *p* < 0.001) compared to non-pregnant participants (Table 5). Among other types of information in which both groups expressed similar interest, information on prevention and control methods related to influenza disease were the most frequently requested (83% vs. 81%, *p* = 0.165). The most sought-after information specifically related to influenza vaccination among pregnant participants was information about possible vaccine side effects (84% vs. 83%, *p* = 0.242) and contraindications to vaccination (83% vs. 86%, *p* = 0.056).

In 2023, there was no significant difference between pregnant and non-pregnant participants in the proportion wanting to receive information related to influenza prevention specifically from a doctor (47% vs. 48%, *p* = 0.573) (Table 5). However, the actual receipt of professional guidance varied between the groups, with a significantly lower proportion of pregnant participants reporting that they received information about influenza prevention from doctors compared to non-pregnant participants (36% vs. 48%, *p* < 0.001).

**Figure 2:**
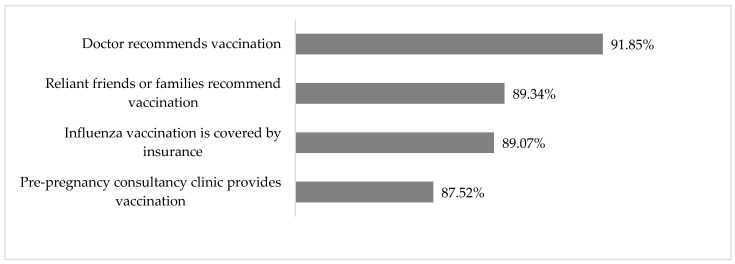
Proportion of individuals who report a “large chance” or “almost certain” chance of being vaccinated against influenza based on the factors listed among pregnant participants. Suzhou, China, 2023.

The acceptance assessment of potential interventions revealed that most pregnant participants (92%) would be more likely to be vaccinated or would almost certainly be vaccinated if a doctor recommended it (Figure 2). Furthermore, over 85% of the pregnant participants believed they would likely be vaccinated if vaccination were provided at no cost (89%), if a friend or family member recommended vaccination (89%), or if vaccination were offered at a pregnancy-planning consultancy clinic (88%).

## 4. Discussions

The cross-sectional surveys conducted in Suzhou, China in 2013 and 2023 revealed distinct patterns of change regarding knowledge specifically related to the influenza vaccine and vaccination services, as well as attitudes toward influenza vaccine effectiveness, willingness to be vaccinated and vaccine uptake between pregnant and non-pregnant participants. There was a notable decline in the awareness of the risks associated with influenza among pregnant participants, with those recognizing severe disease risks dropping from 80% to 62%. Additionally, the rate of willingness to receive the influenza vaccine decreased from 9% to 4%. Horizontally speaking, in 2023, pregnant participants still exhibited lower levels of knowledge about both influenza disease and the influenza vaccine, along with less positive attitudes regarding the effectiveness and safety of the vaccine. They also showed lower willingness to vaccinate and lower rates of vaccine uptake compared to non-pregnant participants.

This study found pregnant participants were rarely vaccinated against influenza in the past decade. The low vaccination-coverage rate among pregnant participants in China was also repeatedly observed in other studies in China [1,13,14,15] and was significantly lower than the global coverage rate of 26% [7]. Similar barriers to increasing vaccination uptake have been repeatedly identified across various studies, including this one; these include concerns about vaccine safety and side effects, as well as doubts about vaccine effectiveness [16]. Additionally, inadequate knowledge about influenza and the influenza vaccine among pregnant women has been frequently observed in countries with low vaccination-coverage rates, as seen in our findings. For instance, a 2024 study in Kyrgyzstan [17] found that only 63% of pregnant women had heard of the influenza vaccine, with a vaccination rate as low as 5.8%. Similarly, a 2023 study in Afghanistan [18] reported that only 56% of pregnant women were aware of the influenza vaccine, resulting in a coverage rate of just 12%. Vaccination coverage in Nicaragua was 16% in 2018 [19], 21% in Thailand in 2016 [20], 32% in Italy in 2017 [21], and 38% in Ecuador in 2021 [22]; these rates are all significantly higher than the near-zero rates observed in our study. These disparities across countries may be attributed to factors such as economic development, cultural differences, and variations in healthcare systems. Moreover, based on the KAP theoretical model [11], one of the reasons for low influenza vaccination coverage among pregnant women may be the lack of these specific knowledge regarding influenza and the influenza vaccine. Misconceptions about the influenza disease and its harm may directly influence their attitude toward the influenza vaccine. Influenza is being confused with other respiratory diseases because patients may not present for health care to be tested for influenza. Patients might assume that they had the flu when in fact they have a different disease. Furthermore, the low perceived risk may also decrease the perceived need for vaccination. In addition, based on the KAP theory, negative attitudes toward the influenza vaccine may further influence their behavior related to influenza vaccination. This was reassured that the concerns regarding influenza vaccine effectiveness and vaccine safety were cited as one of the main reasons for not getting vaccinated among pregnant participants.

Compared to previous KAP-flu studies in China in this population [15], this study highlighted concerning declining trends and provided detailed information on the information that should be included in a campaign to increase seasonal influenza vaccination coverage among pregnant women. The declines in knowledge and attitudes among pregnant women may be attributed to several factors that led to decreased utilization of influenza-vaccination services across all populations in China, including vaccine scandals and changes in vaccine-management policy, such as reduced incentives to administer the influenza vaccine at points of vaccination [23]. Future health-education campaign materials targeting pregnant participants should be tailored to address specific knowledge gaps regarding the disease, including the difference between influenza and the common cold and its potential for harm, and regarding the vaccine, including vaccine safety and effectiveness. Additionally, this study provided important information that revealed differences between pregnant participants and non-pregnant participants in terms of changes that occurred between 2013 and 2023 with regard to KAP-flu in China. Factors such as hormonal changes, heightened health concerns, and societal pressures may influence pregnant participants’ perspectives on influenza vaccination. Therefore, specific mechanisms are warranted to deliver health information especially related to the influenza vaccine.

This study found that most pregnant participants expressed that they would be more likely to be vaccinated or almost certainly would be vaccinated if a doctor recommended it, although pregnant participants were less likely to receive professional information from a doctor compared to non-pregnant participants. Doctors’ important role in increasing vaccination coverage in pregnant participants [20] and other high-risk populations [10] has been previously reported. This study reiterates the importance of a doctor’s recommendation for increasing rates of influenza vaccination during pregnancy. To increase the rate of recommendation by doctors, one strategy would be to provide targeted education equipping doctors with effective messaging. This effort could be combined with public messaging that includes details on vaccination services and policies via popular traditional media or social media.

Considering the intersection of targeted education, promotional strategies, and doctors’ recommendations, leveraging the maternal and child healthcare system to encourage the associated healthcare providers to deliver effective messaging about seasonal influenza vaccination to pregnant women and those considering pregnancy may be a highly effective approach. Initiating these efforts during the pre-pregnancy service period within the maternal and child healthcare system could be particularly feasible in countries where vaccine hesitancy is common in the pregnant population. In the Healthy China Agenda 2019–2030, the National Health Commission included pre-marital and pregnancy planning health checkups along with prenatal healthcare in the performance evaluation for healthcare facilities [24]. By 2019, 76% of women obtained pre-marital healthcare checkups. Leveraging premarital healthcare checkups to provide health education may make it easier for doctors to recommend vaccination, and offering on-site influenza vaccination may help to establish the practice of influenza vaccination among women planning pregnancy and improve their confidence in vaccination in later pregnancy. Moreover, our study found the influenza vaccination coverage rates were higher in family members of pregnant women than in the pregnant women themselves. This result may mean that family members are more inclined to receive vaccines than are pregnant participants. In addition, participants reported they would be more likely to get vaccinated if family members had experiences of safe influenza vaccination. This suggests a supplementary approach of building confidence and influencing pregnant women’s vaccine uptake through their family members; it may thus be beneficial to provide health education to both pregnant participants and to those who accompany them during prenatal care.

This study is subject to at least three limitations. First, the study was conducted in Suzhou, one of the most developed cities in China. The knowledge, attitude and vaccination practices seen in Suzhou may not be generalizable to other areas in China. Our study participants were relatively better educated and of higher economic status compared with the population in other parts of China, and therefore, we expect their knowledge about influenza and their vaccination coverage rates to be higher than in other cities. Secondly, within each level, we selected prenatal-care facilities based on willingness to participate, so these facilities may be systematically different from facilities that were unwilling to participate. In addition, we focused on the KAP theoretical model, which may not fully explain the variance in vaccination practice. There may be other factors related to vaccination that were not evaluated in this study, such as lack of vaccine supply and lack of adult vaccination services.

## 5. Conclusions

Our study revealed significant gaps in knowledge, attitudes, and practices regarding influenza and the influenza vaccine among pregnant women compared to non-pregnant women in Suzhou, China. The results underscore the urgent need for targeted interventions to address misconceptions about the influenza vaccine, particularly within the maternal healthcare system. By enhancing communication strategies and providing clear, evidence-based information, public health initiatives can better support pregnant women in making informed decisions about vaccination, ultimately improving maternal and fetal health outcomes. Effective messaging aimed at increasing influenza-vaccine uptake among pregnant women should clarify the differences between influenza and other respiratory diseases and their implications for vaccine performance. The messaging should address concerns regarding vaccine effectiveness and safety while providing detailed information about vaccination-delivery services, including timing, frequency, and locations.

## Figures and Tables

**Table 1:** Characteristics of pregnant and non-pregnant participants—Suzhou, China, survey in 2013 and survey in 2023.

	Time	Pregnant	Non-Pregnant	*p*-Value
Number of total participants	2013	1673	401	
	2023	2195	1171	
Median age (interquartile range), years	2013	26 (23–29)	24 (23–25)	
	2023	30 (27–33)	29 (25–33)	*0.069*
	*p*-value	***<0.001***	***<0.001***	
Education, college or above	2013	44% (741/1672)	65% (260/399)	
	2023	74% (1632/2195)	88% (1036/1171)	***<0.001***
	*p*-value	***<0.001***	***<0.001***	
Household annual income ≥ CNY 250,000	2013	3% (47/1666)	5% (21/401)	
	2023	27% (591/2195)	19% (219/1171)	***<0.001***
	*p*-value	***<0.001***	***<0.001***	
Median number of other persons living in the household (range)	2013	3 (1–10)	2 (1–6)	
	2023	2 (0–7)	2 (0–8)	***<0.001***
	*p*-value	***<0.001***	***<0.001***	
Presence of underlying health condition	2013	2.3% (39/1660)	0.3% (1/399)	
	2023	2.6% (56/2195)	2.4% (28/1171)	*0.867*
	*p*-value	*0.768*	*0.012*	
BMI before pregnancy, mean ± standard deviation	2013	20.9 ± 3.28	20.3 ± 2.48	
	2023	22.4 ± 3.72	21.9 ± 3.86	***<0.001***
	*p*-value	***<0.001***	***<0.001***	

Data represented as % (n/N) unless otherwise as indicated; n is the number of persons in the numerator, and N is the total number of persons with data for each group.

**Table 2:** Knowledge and attitudes regarding influenza and the influenza vaccine among pregnant and non-pregnant participants—Suzhou, China, survey in 2013 and survey in 2023.

	Time	Pregnant	Non-Pregnant	*p*-Value
**Knowledge of influenza**				
Know the main symptoms of influenza, including both fever and cough	2013	91% (1525/1673)	88% (351/401)	
	2023	95% (2093/2195)	98% (1142/1171)	***0.003***
	*p*-value	***<0.001***	***<0.001***	
Know at least one of the routes by which influenza spreads, including coughing and indirect contact through hands	2013	98% (1640/1673)	96% (384/401)	
	2023	99% (2184/2195)	100% (1169/1171)	*0.238*
	*p*-value	***<0.001***	***<0.001***	
Know at least one of the measures used to prevent influenza, including vaccination and non-pharmaceutical interventions	2013	97% (1619/1673)	85% (342/401)	
	2023	99% (2177/2195)	99% (1162/1171)	*1.000*
	*p*-value	***<0.001***	***<0.001***	
Know that influenza and colds are different diseases	2013	35% (583/1673)	37% (148/401)	
	2023	49% (1076/2195)	42% (495/1171)	***<0.001***
	*p*-value	***<0.001***	*0.068*	
Influenza can cause severe complications	2013	80% (1335/1673)	92% (368/401)	
	2023	62% (1351/2195)	77% (899/1171)	***<0.001***
	*p*-value	***<0.001***	***<0.001***	
Influenza can cause hospitalization	2013	75% (1256/1673)	92% (367/401)	
	2023	65% (1427/2195)	78% (917/1171)	***<0.001***
	*p*-value	***<0.001***	***<0.001***	
Influenza can cause death	2013	63% (1059/1673)	73% (294/401)	
	2023	49% (1079/2195)	70% (824/1171)	***<0.001***
	*p*-value	***<0.001***	*0.289*	
Influenza during pregnancy may result in miscarriage/stillbirth	2013	13% (213/1673)	3% (11/401)	
	2023	44% (969/2195)	52% (604/1171)	***<0.001***
	*p*-value	***<0.001***	***<0.001***	
Influenza during pregnancy may lead to abnormal fetal development	2013	28% (473/1673)	22% (88/401)	
	2023	50% (1095/2195)	55% (643/1171)	***0.006***
	*p*-value	***<0.001***	***<0.001***	
**Knowledge of the influenza vaccine**				
Self-report “ever heard” of the influenza vaccine	2013	56% (939/1673)	55% (222/401)	
	2023	57% (1261/2195)	78% (914/1171)	***<0.001***
	*p*-value	*0.430*	***<0.001***	
***The data below represent those who ever heard of the influenza vaccine***				
Know to get the influenza vaccine annually	2013	39% (366/939)	49% (109/222)	
	2023	33% (421/1261)	41% (373/914)	***<0.001***
	*p*-value	***0.043***	***0.030***	
Know September–November is the time to get the influenza vaccine in Suzhou	2013	14% (134/939)	6% (14/222)	
	2023	12% (153/1261)	20% (180/914)	***<0.001***
	*p*-value	***0.008***	***<0.001***	
Know that pregnant women or those planning pregnancy constitute a priority group who should receive the influenza vaccine	2013	12% (109/939)	6% (13/222)	
	2023	20% (257/1261)	25% (226/914)	***0.019***
	*p*-value	***<0.001***	***<0.001***	
Know community health centers are the location for influenza vaccination in Suzhou	2013	34% (317/939)	10% (22/222)	
	2023	66% (832/1261)	73% (663/914)	***<0.001***
	*p*-value	***<0.001***	***<0.001***	
**Attitude regarding influenza vaccination**				
Think the influenza vaccine works well or works sometimes	2013	91% (851/939)	84% (186/222)	
	2023	76% (960/1261)	82% (750/914)	***0.001***
	*p*-value	***<0.001***	*0.612*	
Think the influenza vaccine is safe or very safe	2013	64% (601/939)	64% (142/222)	
	2023	69% (867/1261)	81% (744/914)	***<0.001***
	*p*-value	***0.022***	***<0.001***	

Data represented as % (n/N) unless otherwise as indicated; n is the number of persons in the numerator (the number who answered the question correctly), and N is the total number of persons with data for each group.

**Table 3:** Influenza-prevention practices among pregnant and non-pregnant participants—Suzhou, China, survey in 2013 and survey in 2023.

	Time	Pregnant	Non-Pregnant	*p*-Value	
Survey respondent vaccinated with the influenza vaccine in the past 12 months	2013	0.6% (10/1673) *	0.0% (0/401)		
	2023	1.9% (42/2195) *	5.3% (62/1171)	***<0.001***	
	*p*-value	*0.001* *	***<0.001***		
Family members vaccinated with the influenza vaccine in the past 12 months	2013	8% (127/1673)	1% (5/401)		
	2023	7% (150/2195)	13% (147/1171)	***<0.001***	
	*p*-value	*0.400*	***<0.001***		
Large chance of being willing or almost certainly willing to receive an influenza vaccine while pregnant (among participants who had ever heard of the influenza vaccine)	2013	9% (87/939)	3% (7/222)		
	2023	4% (45/1261)	9% (86/914)	***<0.001***	
	*p*-value	***<0.001***	***0.004***		
**Among participants with a family member who had a fever and cough**					
Try to avoid close contact with patient	2013	25% (86/349)	11% (10/92)		
	2023	87% (680/781)	88% (462/527)	*0.815*	
	*p*-value	***<0.001***	***<0.001***		
Increase hand-washing frequency	2013	10% (36/349)	0% (0/92)		
	2023	76% (591/781)	76% (402/527)	*0.852*	
	*p*-value	***<0.001***	***<0.001***		
Wear a mask	2013	6% (22/349)	0% (0/92)		
	2023	82% (637/781)	81% (427/527)	*0.863*	
	*p*-value	***<0.001***	***<0.001***		

* All vaccinated pregnant participants had been administered the influenza vaccine prior to their pregnancy.

**Table 4:** Perceived “very relevant” or “extremely relevant” reasons to receive or not receive influenza vaccination among pregnant participants who had ever heard of the influenza vaccine—Suzhou, China, 2023.

	Percentage (n/N)
**Perceived “very relevant” or “extremely relevant” reasons to receive an influenza vaccination**	
I get sick with the flu more easily than other people my age.	17 (213/1261)
My doctor has recommended that I get a flu vaccination before or during my pregnancy.	**27 (342/1261)**
I am worried that flu will bring harm to my fetus.	**36 (455/1261)**
I think flu is high-risk for pregnant participants.	**29 (360/1261)**
It is good for my family members.	12 (148/1261)
**Perceived “very relevant” or “extremely relevant” reasons not to receive an influenza vaccination**	
I had a severe reaction following a prior vaccination.	**20 (245/1219)**
I had concerns about side effects.	**15 (187/1219)**
I had concerns about getting the flu from the flu shot.	9 (108/1219)
I think flu vaccines do not work.	9 (112/1219)
Flu vaccination is not needed.	9 (107/1219)
I’m allergic to the vaccine.	**20 (238/1219)**
Flu is not a very serious illness.	11 (131/1219)
I do not have chances to have contact with people who get the flu.	8 (97/1219)
I had had the flu earlier in the season.	8 (100/1219)

**Table 5:** The knowledge and information participants want to receive and favorable media—Suzhou, China, 2023.

	Pregnant N = 2195	Non-Pregnant N = 1171	*p*-Value
**What influenza-related knowledge and information do you want?**			
Prevention and control methods	83 (1817)	81 (946)	*0.165*
Transmission route	80 (1759)	78 (913)	*0.151*
Harm	77 (1690)	78 (916)	*0.441*
Characteristics of transmission	76 (1673)	77 (898)	*0.794*
Treatment methods	71 (1567)	73 (860)	*0.221*
**What influenza vaccine-related knowledge and information do you want to receive?**			
Side effects	84 (1852)	83 (969)	*0.242*
Vaccination contraindications	83 (1829)	86 (1006)	*0.056*
Effectiveness of the vaccine	77 (1682)	77 (902)	*0.827*
Vaccination time and location	**73 (1607)**	**78 (910)**	***0.005***
Vaccination policy (e.g., priority populations)	**51 (1130)**	**59 (687)**	***<0.001***
**How do you hope to access knowledge about flu prevention?**			
Networks	**79 (1744)**	**84 (978)**	***0.005***
TV	**55 (1215)**	**62 (721)**	***0.001***
Doctor	47 (1039)	48 (567)	*0.573*
Family/friends	37 (807)	38 (442)	*0.601*
Broadcast	**35 (772)**	**47 (552)**	***<0.001***
Newspapers/magazines	**27 (589)**	**37 (434)**	***<0.001***
Billboards/manuals	**24 (526)**	**30 (349)**	***<0.001***
Others	1 (16)	1 (9)	*1.000*
**How do you access knowledge about flu prevention?**			
Networks, Internet	78 (1718)	81 (948)	*0.074*
TV	**63 (1381)**	**69 (806)**	***0.001***
Family/friends	51 (1121)	51 (598)	*1.000*
Doctor	**36 (797)**	**48 (559)**	***<0.001***
Broadcast	**35 (766)**	**49 (574)**	***<0.001***
Newspapers/magazines	**24 (534)**	**39 (455)**	***<0.001***
Billboards/manuals	**18 (396)**	**24 (283)**	***<0.001***
Others	1 (22)	1 (7)	*0.311*

Data are represented as % (n).

## Data Availability

The data presented in this study are available on request from the corresponding author due to ethical reasons.

## Supplementary Materials

The following supporting information can be downloaded at: https://www.mdpi.com/article/10.3390/vaccines13060589/s1, Supplement S1: Knowledge, attitude and practice items in the questionnaire; Supplement S2: Regression Results of DID Model (*point*) on Knowledge.

## References

[1] Sun J., Zhang Y., Zhou S., Song Y., Zhang S., Zhu J., Zhu Z., Wang R., Chen H., Chen L. (2024). Laboratory-confirmed influenza hospitalizations during pregnancy or the early postpartum period—Suzhou City, Jiangsu Province, China, 2018–2023. MMWR. Morb. Mortal. Wkly. Rep..

[2] Wang R., Yan W., Du M., Tao L., Liu J. (2021). The effect of influenza virus infection on pregnancy outcomes: A systematic review and meta-analysis of cohort studies. Int. J. Infect. Dis..

[3] Fell D., Savitz D., Kramer M., Gessner B., Katz M., Knight M., Luteijn J., Marshall H., Bhat N., Gravett M. (2016). Maternal influenza and birth outcomes: Systematic review of comparative studies. BJOG Int. J. Obstet. Gynaecol..

[4] Thompson M.G., Kwong J.C., Regan A.K., Katz M.A., Drews S.J., Azziz-Baumgartner E., Klein N.P., Chung H., Effler P.V., Feldman B.S. (2019). Influenza Vaccine Effectiveness in Preventing Influenza-associated Hospitalizations During Pregnancy: A Multi-country Retrospective Test Negative Design Study, 2010–2016. Clin. Infect. Dis. Off. Publ. Infect. Dis. Soc. Am..

[5] Irving S.A., Ball S.W., Booth S.M., Regan A.K., Naleway A.L., Buchan S.A., Katz M.A., Effler P.V., Svenson L.W., Kwong J.C. (2021). A multi-country investigation of influenza vaccine coverage in pregnant individuals, 2010–2016. Vaccine.

[6] Hirve S., Lambach P., Paget J., Vandemaele K., Fitzner J., Zhang W. (2016). Seasonal influenza vaccine policy, use and effectiveness in the tropics and subtropics—A systematic literature review. Influenza Other Respir. Viruses.

[7] Chen C., Liu X., Yan D., Zhou Y., Ding C., Chen L., Lan L., Huang C., Jiang D., Zhang X. (2022). Global influenza vaccination rates and factors associated with influenza vaccination. Int. J. Infect. Dis..

[8] Chinese Center for Disease Control and Prevention (2023). Technical guidelines for seasonal influenza vaccination in China (2023–2024). Zhonghua Liu Xing Bing Xue Za Zhi.

[9] Chinese Center for Disease Control and Prevention (2010). Technical Guidelines for the Application of Seasonal Influenza Vaccine in China (2010–2011).

[10] Song Y., Zhang T., Chen L., Yi B., Hao X., Zhou S., Zhang R., Greene C. (2017). Increasing seasonal influenza vaccination among high risk groups in China: Do community healthcare workers have a role to play?. Vaccine.

[11] Green L., Kreuter M. (2005). Green LW, Kreuter MW. Health Program Planning: An Educational and Ecological Approach.

[12] Henninger M.L., Irving S.A., Thompson M., Avalos L.A., Ball S.W., Shifflett P., Naleway A.L. (2015). Factors associated with seasonal influenza vaccination in pregnant women. J. Women’s Health (2002).

[13] Su L., Chen H., Zhou X., Zhang K., Pan C. (2018). Seasonal Influenza Vaccination and influencing Factors in 2417 Pregnant Women in Shenzhen. J. Prev. Med. Chin. People’s Lib. Army.

[14] Chen L., Zhou S., Bao L., Millman A.J., Zhang Z., Wang Y., Tan Y., Song Y., Cui P., Pang Y. (2022). Incidence rates of influenza illness during pregnancy in Suzhou, China, 2015–2018. Influenza Other Respir. Viruses.

[15] Li R., Xie R., Yang C., Rainey J., Song Y., Greene C. (2018). Identifying ways to increase seasonal influenza vaccine uptake among pregnant women in China: A qualitative investigation of pregnant women and their obstetricians. Vaccine.

[16] Nowak G.J., Sheedy K., Bursey K., Smith T.M., Basket M. (2015). Promoting influenza vaccination: Insights from a qualitative meta-analysis of 14 years of influenza-related communications research by U.S. Centers for Disease Control and Prevention (CDC). Vaccine.

[17] Akmatova R., Dzhangaziev B., Ebama M.S., Otorbaeva D. (2024). Knowledge, attitudes, and practices (KAP) towards seasonal influenza and influenza vaccine among pregnant women in Kyrgyzstan: A cross-sectional study. Vaccine.

[18] Shahid S., Kalhoro S., Khwaja H., Hussainyar M.A., Mehmood J., Qazi M.F., Abubakar A., Mohamed S., Khan W., Jehan F. (2023). Knowledge, attitudes, and practices towards seasonal influenza vaccination among pregnant women and healthcare workers: A cross-sectional survey in Afghanistan. Influenza Other Respir. Viruses.

[19] Arriola C.S., Vasconez N., Bresee J., Ropero A.M. (2018). Knowledge, attitudes and practices about influenza vaccination among pregnant women and healthcare providers serving pregnant women in Managua, Nicaragua. Vaccine.

[20] Ditsungnoen D., Greenbaum A., Praphasiri P., Dawood F.S., Thompson M.G., Yoocharoen P., Lindblade K.A., Olsen S.J., Muangchana C. (2016). Knowledge, attitudes and beliefs related to seasonal influenza vaccine among pregnant women in Thailand. Vaccine.

[21] Napolitano F., Napolitano P., Angelillo I.F. (2017). Seasonal influenza vaccination in pregnant women: Knowledge, attitudes, and behaviors in Italy. BMC Infect. Dis..

[22] Erazo C.E., Erazo C.V., Grijalva M.J., Moncayo A.L. (2021). Knowledge, attitudes and practices on influenza vaccination during pregnancy in Quito, Ecuador. BMC Public Health.

[23] Zhou S., Greene C.M., Song Y., Zhang R., Rodewald L.E., Feng L., Millman A.J. (2019). Review of the status and challenges associated with increasing influenza vaccination coverage among pregnant women in China. Hum. Vaccines Immunother..

[24] The General Office of the State Council There Are More Than 4000 Prenatal Screening Institutions, and China’s Birth Defect Prevention and Treatment Network Is Constantly Improving. https://www.gov.cn/yaowen/liebiao/202407/content_6963680.htm.

