# Supplementary material for: Opportunities to Increase Influenza Vaccine Uptake Among Pregnant Women: Insights from Surveys in 2013 and 2023
Source: Vaccines (Basel). 2025 May 30;13(6):589. doi: 10.3390/vaccines13060589 (PMC12197754; doi:10.3390/vaccines13060589)
Supplement: Supplementary file 1 [file vaccines-13-00589-s001.zip › vaccines-3632770-supplementary.pdf]

Supplement S1
Knowledge, attitude and practice items in the questionnaire
B1 How do you think about the difference and relationship of flu and cold?
①Same disease ②Cold is one kind of flu ③Flu is one kind of cold ④Different disease ⑤Other_________
B2 What pathogen cause influenza?
①Pathogen (bacteria, virus, etc.) ②Be affected by cold or heat ③Eating disprders/bad rest ④Do not know
B3 Do you know the main symptoms of influenza? (Multiple choices) (Do not read the list. Check off ones that the person mentions)
①Fever ②Cough ③Sore throat ④ Nasal congestion, runny nose ⑤Muscle or Body aches
⑥Headache ⑦ Fatigue ⑧Vomit, diarrhea ⑨Do not know
B4 Perception about seriously bad outcome:
B4.1 Can flu cause severe complications? ①Yes ②No ③Do not know
B4.2 Can flu cause hospitalization? ①Yes ②No ③Do not know
B4.3 Can flu cause death? ①Yes ②No ③Do not know
B5 Do you think it will be harm to your baby if you got flu during pregnancy? (Multiple choices) (Do not read the list. Check off ones that the person mentions)
①Result in miscarriage/stillbirth ②Lead to dysplasia ③Harms, but don't know details ④No harms ⑤ Other_________
B6 Do you know the routes of infecting or spreading influenza? _________ (Multiple choice) (Do not read the list. Check off ones that the person mentions)
①Coughing, sneezing and talking closely ②In an unventilated environment ③Shake hands, hug
④Indirect contact through hand (such as door handle) ⑤Food ⑥Other________
B7 Do you know some of the measures preventing influenza? (Multiple choices) (Do not read the list. Check off ones that the person mentions)
B7.1 Influenza vaccination
B7.2 Washing hands frequently by water and soap
B7.3 Avoiding crowds
B7.4 Wearing mask when you go to crowded place
B7.5 Avoiding contacting with flu patients (or people get a cough) closely
B7.6 Ventilating the indoor air and cleaning room
B7.7 Taking some drugs for prevention
B7.8 Others
B8 What time do people more likely to get flu in Suzhou?
①All year round ②Winter-spring ③Spring-summer ④Summer-autumn ⑤Autumn-Winter ⑥Do not know
C1 Are you worried that the family will be suffering from influenza?
(1)Not worried (2)A little worried (3)Very worried (4)Never concern
C2 In the past three months, have your family members (living in the same house with you) gotten the symptoms of fever and cough?
①Yes (relationship . And answer C2.1 and C2. ②No ③Do not know
C2.1 What did patient do if yes? (Multiple choices)
①Go to hospital②No treatment ③Self-medication ④Rest at home and avoiding contact with others
⑤Wearing a mask when going out ⑥Others ⑦No interventions
C2.2 What did others do for prevention if yes?
①Try to avoid close contact with their families and others ②Increase hand-washing times ③Wearing mask
④Maintain good indoor ventilation ⑤Taking some drugs for prevention
⑥Other ⑦No preventions
D1 Do you know there has vaccine against influenza? (If ② or ③, go to Part 6)
①Yes ②No ③Do not know
D2 Do you know the right time to get influenza vaccine in Suzhou?
①March-May ②June -August ③September-November ④December-February
⑤Other ⑥Do not know
D3 Do you know the influenza vaccination location in Suzhou? (Multiple answer)
①Hospitals ②CDC ③Community health centers ④Other ⑤Do not know
D4 Do you know how often you need to get the influenza vaccine?
①Only one dose for whole life ②One dose every 5 years or more ③One dose every 2-5 year
④One dose every year ⑤Do not know

D5 Do you know who should take the influenza vaccine first? (Multiple answers) (Do not read the list. Check off ones that the person mentions)
①Children less than 5 years old ②People old than 50 years old
③Patients with chronic diseases ④Woman being or be ready to pregnant
⑤Health Workers ⑥Do not know ⑦Other
E1 Do you think influenza vaccine is safe?
①It's very safe ②It's safe ③It's not so safe ④It's not safe ⑤Do not know
E2 What do you think about the possible adverse reactions after flu vaccination?
①Because of adverse reactions so I don't want to vaccination
②The adverse reactions is rare, so vaccination is necessary
③I just don't want to vaccination no matter of the adverse reactions
④Other
E3 Do you think flu vaccine works?
①Works well ②Works sometimes ③Invalid ④Do not know
E4 What are the chances that you would agree to get the flu vaccination?
(1= Almost zero chance 2=Small chance 3=Moderate chance 4= Large chance 5= Almost certain to get vaccinated)
E4.1 While you are pregnant:
E4.2 While you are not pregnant:
E5 In The past year, have your family vaccinated with influenza vaccine?
①Yes (relationship ______________) ②No ③Not clear
E6.In the past year, have you vaccinated with the influenza vaccine?
①Yes (Time: yyyy_____mm_____) (go to E8) ②No
E7 There are many reasons why people don't get flu vaccinations. For each of the following, rate how relevant this reason was to you. (1=Not at all 2=A little relevant to me 3=Somewhat relevant 4=Very relevant to me 5=Extremely relevant to me)
E7.1 I had a severe reaction following a prior vaccination
E7.2 I had concerns about side effects
E7.3 I had concerns about getting the flu from the flu shot
E7.4 I think flu vaccines do not work
E7.5 Flu vaccination is not needed
E7.6 I'm allergic to the vaccine
E7.7 Flu is not a very serious illness
E7.8 I do not have chance to contact with people who get the flu
E7.9 I had already had flu earlier in the season
E7.10 Other
(E7.11-E7.13 are only for pregnant women)
E7.11 My doctor has discouraged me from getting a flu vaccination while pregnant .
E7.12 Friends or family members have discouraged me from getting a flu vaccination while pregnant
E7.13 I 'm worried that flu vaccine maybe harm for my fetus.
E8 There are a variety of reasons why people get an influenza vaccination. For each of the following, rate how relevant this reason was to you? (1=Not at all 2=A little relevant to me 3=Somewhat relevant 4=Very relevant to me 5=Extremely relevant to me)
E8.1 I get sick with the flu more easily than other people my age.
E8.2 My doctor has recommended that I get a flu vaccination before or during my pregnancy.
E8.3 I am worried that flu will bring harm to my fetus
E8.4 I think flu is a high risk factor to pregnant women
E8.5 Good for my family members
E8.6 Others
E9 For selecting the influenza vaccine, what is your first concern?
①Price ②Safety ③Effectiveness ④The place of origin (imported first)
Please sort all options above: \ \ \

Supplement S2

Regression Results of DID Model (*point*) on Knowledge

Variable	(1) *point* β ($s_{\bar{x}}$)	(2) *point* β ($s_{\bar{x}}$)	(3) *point* β ($s_{\bar{x}}$)	(4) *point* β ($s_{\bar{x}}$)	(5) *point* β ($s_{\bar{x}}$)	(6) *point* β ($s_{\bar{x}}$)	(7) *point* β ($s_{\bar{x}}$)	(8) *point* β ($s_{\bar{x}}$)
pt	-1.449*** (0.180)	-1.307*** (0.180)	-1.319***(0.180)	-1.305*** (0.180)	-1.417*** (0.172)	-1.541*** (0.172)	-1.582*** (0.173)	-1.575*** (0.172)
p	-0.001 (0.151)	-0.168 (0.151)	-0.156 (0.151)	-0.161 (0.151)	0.236 (0.145)	0.381*** (0.147)	0.402*** (0.147)	0.406*** (0.147)
t	2.738*** (0.157)	2.355*** (0.162)	2.389*** (0.163)	2.311*** (0.165)	1.884*** (0.158)	1.948*** (0.158)	1.913*** (0.158)	1.903*** (0.158)
age		0.070*** (0.008)	0.077*** (0.009)	0.076*** (0.009)	0.043*** (0.009)	0.040*** (0.009)	0.036*** (0.009)	0.033*** (0.009)
parturition			-0.126* (0.068)	-0.119* (0.067)	0.281*** (0.067)	0.302*** (0.067)	0.294*** (0.067)	0.288*** (0.067)
residence				0.471*** (0.124)	0.176 (0.119)	0.177 (0.118)	0.155 (0.118)	0.152 (0.118)
education					1.202*** (0.053)	1.067*** (0.057)	1.001*** (0.059)	0.970*** (0.060)
job						0.370*** (0.060)	0.344*** (0.061)	0.307*** (0.062)
income							0.256*** (0.067)	0.248*** (0.067)
insurance								0.412*** (0.113)
Constant	6.200*** (0.136)	4.505*** (0.237)	4.336*** (0.254)	3.882*** (0.280)	2.314*** (0.276)	3.499*** (0.337)	3.177*** (0.347)	3.747*** (0.380)
R^2	0.125	0.137	0.137	0.140	0.216	0.222	0.224	0.226

Note: ***α=0.01, **α=0.05, *α=0.1
